# Knowledge and attitude of pregnant women about preeclampsia in King Abdulaziz Medical City, Western Region: A cross-sectional study

**DOI:** 10.1371/journal.pone.0312304

**Published:** 2025-05-07

**Authors:** Fatimah Ahmed Alsabi, Abeer Mokhtar Orabi, Eman Zain Bajamal

**Affiliations:** 1 College of Nursing, King Saud bin Abdulaziz University for Health Sciences, Jeddah, Saudi Arabia; 2 King Abdullah International Medical Research Center, Jeddah, Saudi Arabia; Innovative Aid, CANADA

## Abstract

**Introduction:**

Preeclampsia is a serious obstetrics condition generally diagnosed by the presence of high blood pressure (≥140/90 mmHg) and significant protein in urine (≥ 2++ on dipstick urine test) developing for the first time after 20 weeks of pregnancy. It poses severe risks to both mother and newborn, including the potential for maternal and neonatal mortality and morbidity. Although preeclampsia is a well-recognized pregnancy-related complication, there is a notable lack of knowledge and awareness among pregnant women in Saudi Arabia, particularly concerning its signs, symptoms, risk factors, and the importance of timely medical intervention. This study aims to assess the level of knowledge and attitude regarding preeclampsia among pregnant women attending King Abdulaziz Medical City in Jeddah, Saudi Arabia.

**Methods:**

In this quantitative cross-sectional study, 122 pregnant women visiting the Obstetrics and Gynecology Outpatient Department were surveyed from April to June 2023. The research employed a structured questionnaire to collect detailed information on participants’ sociodemographic backgrounds, knowledge about preeclampsia symptoms, risk factors, complications, and attitudes toward its prevention and the recognition of danger signs. Data were analyzed using descriptive statistics as well as ANOVA test, T-test, and Pearson correlation to explore relationships and significance among study variables.

**Results:**

The study revealed an average knowledge score of 25.43 (SD = 5.67), indicating that many participants demonstrated a moderate understanding of preeclampsia. On the other hand, the attitude score averaged 23.39 (SD = 3.11), showing that participants held favorable attitudes toward the risk factors, prevention, and complications of preeclampsia. Notably, participants with higher education levels and those who were employed demonstrated higher knowledge scores.

**Conclusion:**

The findings reflect a moderate level of understanding among participants but also highlight critical areas where knowledge enhancement is necessary to enable timely intervention and reduce adverse maternal and neonatal outcomes.

## Introduction

Preeclampsia is a multisystemic pregnancy disorder characterized by high blood pressure (≥140/90 mmHg) and the presence of significant protein in urine (≥ 2++ on dipstick urine test) developing for the first time after 20 weeks of pregnancy. This condition has been documented to significantly decrease blood supply to the fetus, thereby causing preterm labor or low birth weight. In severe cases, it can escalate to eclampsia, which involves the occurrence of seizures or coma, demanding immediate medical intervention to prevent fatal outcomes [1,2]. The global incidence of preeclampsia was estimated at 4.6% in a systematic review by Abalos et al. [3]. A more recent population-based study by Xie et al. [4] observed a 10.92% increase in the incidence from 1990 to 2019, indicating a rising trend in cases worldwide. On a national scale, detailed research done by Subki et al. [5] evaluated the medical records from Jeddah between January 2015 and June 2017 and identified a high prevalence and significant complications associated with hypertensive disorders, including preeclampsia. Further reports from the Saudi Ministry of Health [6] documented that in 2018, approximately 0.7% of pregnancies (4,372 out of 624,580) were complicated by preeclampsia, with 12.1% of these cases developing into eclampsia. In 2021, about 0.65% of pregnancies (4,004 out of 617,750) were affected by preeclampsia, with 11.2% of these progressing to eclampsia. These statistics underscore the ongoing challenge and highlight the critical need for enhanced preventive strategies and robust healthcare interventions to manage this condition effectively within the kingdom. The World Health Organization recognizes preeclampsia as a preventable complication of pregnancy that can lead to maternal mortality [7]. Mental and occupational stress, depression, and anxiety have been associated with higher incidences of the condition. Understanding and mitigating the risk factors through targeted interventions such as stress management, dietary adjustments, and regular antenatal visits can significantly lower the risk of developing preeclampsia [8,9]. Attitudes toward preeclampsia prevention and recognizing its danger signs are also crucial in effectively managing this condition. In this regard, a study by Crombag et al. [10] highlighted a generally positive attitude toward preeclampsia screening and follow-up among pregnant women, underscoring the importance of continuous education and support provided by healthcare professionals, particularly midwives, and nurses, in fostering a proactive approach to managing preeclampsia. This is compounded by the findings by Jama [11] in Somalia which revealed that only 40% of pregnant women would seek medical attention upon experiencing symptoms of preeclampsia, suggesting a gap in awareness and proactive health-seeking behavior [11–13]. Despite these efforts, a substantial knowledge gap exists among pregnant women regarding preeclampsia. Recent studies across different regions have shown low levels of awareness and understanding of the condition, its symptoms, and the necessary preventive measures. In this perspective, a study in Makkah found that only 4% of participants had adequate knowledge about preeclampsia [14]. Similarly, research in Ghana revealed that one-third of postpartum women treated for preeclampsia had not received any counseling from healthcare providers regarding their condition during pregnancy, with more than half remaining uninformed about prevention in future pregnancies [15]. These findings indicate a critical need for standardized education on preeclampsia early in pregnancy to improve maternal and fetal outcomes. Hansson et al. [16] emphasized the necessity of such education, particularly for nulliparous women who often struggle to distinguish between normal pregnancy symptoms and those of preeclampsia. Similarly, Alnuaimi et al. [17] highlight the significant impact that increased awareness and educational interventions can have on preventing and managing preeclampsia. In the region where the study was conducted, some group education sessions are held, particularly for first-time mothers, where they are informed about pregnancy-related conditions, including preeclampsia. However, these educational efforts are often limited to those who regularly attend antenatal care appointments. In conclusion, enhancing the knowledge and attitudes of pregnant women toward preeclampsia through comprehensive educational programs and robust healthcare provider engagement is vital. Such initiatives could significantly reduce the incidence of preeclampsia and its associated complications, thereby decreasing maternal and neonatal morbidity and mortality. This study seeks to identify gaps in knowledge and attitude that could inform targeted educational interventions and health policy adjustments.

## Materials and methods

### Research design and setting

A quantitative cross-sectional research design was employed to evaluate the knowledge and attitudes of pregnant women regarding preeclampsia. The study was conducted at the Obstetrics and Gynecology Outpatient Department of King Abdul-Aziz Medical City in the Western Region of Saudi Arabia. It is a governmental tertiary hospital offering medical care services for the Saudi National Guard personnel and their families in the western area. The average annual antenatal care attendance in the hospital is about 9500.

### Sample and sampling

The study population comprised pregnant women attending the outpatient department who met the inclusion criteria and consented to participate. Eligible participants were those aged 18–45 years who were literate to ensure that participants could independently understand and respond to the questions, which were critical in gathering accurate data on their knowledge and attitudes toward preeclampsia. Sample size determination utilized G*Power 3.1 software through a power analysis. To achieve a 95% confidence interval within 5% of the true value, the required sample size was set at 122. The input data were a population size of 9500 in the last year. Convenience sampling was employed to recruit participants who met the inclusion criteria and were available during the study period.

### Instrument and data collection

Data for this study were gathered from April to June 2023 using a structured questionnaire developed from a synthesis of research published in the literature [17–19]. To access the questionnaire, participants scanned a barcode, which provided an autonomous consent form and the questionnaire on their mobile devices. For those unable to use their phones, devices were made available by the research team. The questionnaire was divided into three distinct sections: demographic characteristics, knowledge, and attitudes toward preeclampsia. The demographic section was split into two parts; the first gathered basic information such as age, educational level, occupation, residence, cigarette smoking habits, weight, and height. The second part collected data on medical and obstetric history, including gestational age, gravidity, number of children, previous history of preeclampsia, family history of preeclampsia, and the number of antenatal care visits during the current pregnancy. The knowledge section comprised 19 items covering symptoms, risk factors, and complications of preeclampsia. Responses were collected using a three-point Likert scale: 2 for true, 1 for I don’t know, and 0 for false. The final section on attitudes was divided into two parts: the first assessed reactions to preeclampsia danger signs such as epigastric pain, diminished fetal movement, vaginal bleeding, and blurred vision, with response options of seeking medical care, doing nothing, or self-medicating. While these responses provided valuable insights into behavior, they were not factored into the computed attitudes score. The second part contained nine items relating to risk factors, prevention, and complications of preeclampsia, also rated on a three-point Likert scale (3 for agree, 2 for neutral, 1 for disagree). Only this section was used to calculate the overall attitudes score, reflecting respondents’ perceptions of preeclampsia-related factors. To ensure the accuracy and consistency of the questionnaire across languages, the survey was translated from English into simple Arabic and then back-translated into English by professional translation services. This process verified that the questions remained consistent in both language versions. To establish content validity, the questionnaire was reviewed by a panel of experts in maternity and community nursing. These experts assessed the survey items for clarity and relevance to the study’s objectives, ensuring that the questionnaire accurately reflected the research themes. The reliability of the questionnaire was verified through the calculation of the Cronbach alpha coefficient. This statistical measure was used to determine the internal consistency of the questionnaire. A pilot study was conducted with 22 pregnant women who represented 20% of the intended study participants who met all the inclusion criteria. The purpose of the pilot study was to test the completeness, accuracy, and clarity of the questionnaire. Results from this preliminary assessment indicated Cronbach’s Alpha of 0.72 for both the knowledge and attitude scales, which was deemed acceptable. After completing the questionnaire, participants were informed about key aspects of preeclampsia that they might not have been aware of. This included the importance of recognizing early symptoms and the need for prompt medical attention. Participants were also given resources on where to seek further information and guidance, emphasizing the critical role of early detection and management in reducing the risks associated with preeclampsia.

### Data analysis

The data collected in this study were entered, coded, and analyzed using the International Business Machines-Statistical Package for Social Sciences (IBM-SPSS version 29). Descriptive statistics were then computed, which included the calculation of frequencies, percentages, means, and standard deviations, providing a quantitative summary of the data. To evaluate the normality of the data distribution, the Shapiro-Wilk test was applied, along with visual assessments using SPSS-generated charts. For inferential statistics, the study employed ANOVA test, and T-test to explore the relationships and significance among the study variables. Additionally, Pearson correlation coefficients were calculated to measure the strength and direction of associations between continuous variables. Statistical significance was determined at a P-value of ≤0.05.

### Ethical considerations

Ethical approval for this study was secured before commencement, from the College of Nursing Research Unit, the Institutional Review Board at King Abdulaziz Medical City-Western Region, and the King Abdullah International Medical Research Center in Jeddah, Saudi Arabia. All participants who met the inclusion criteria were required to sign a consent form before participating in the study. This form outlined the voluntary nature of their participation, clearly stating that they could refuse to participate or withdraw from the study at any time without any consequences. To safeguard participant privacy and confidentiality, all collected data were stored electronically on a password-protected computer. Access to this data was limited to the research team.

## Results

The present study included 122 pregnant women with an average age of 30.89 years (SD = 6.21). Their average weight and height were 68.85 kg (SD = 12.46) and 156.46 cm (SD = 5.74). All the participants (100%) were married. More than half of the participants (57.4%) had a university education. About (80.3%) were housewives, while (12.3%) worked in the non-healthcare sector. Most of the participants, (86.9%), were urban residents. Only (0.8%) of them reported smoking, while (99.2%) were non-smokers (Table 1).

**Table 1:** Sociodemographic Characteristics of the Participants.

Variables	*Mean*	*SD*	
Age	30.89	6.215	
Weight (kg)	68.85	12.461	
Height (cm)	156.46	5.743	
BMI	28.798	7.556	
**Variables**		***N***	***%***
BMI	Underweight	4	3.3
	Proper weight	28	23.0
	Overweight	54	44.3
	Obesity	36	29.5
Marital status	Married	122	100
Level of education	High school or less	45	36.9
	University education	70	57.4
	Postgraduate education	7	5.7
Employment sector	Health sector	9	7.4
	Non-health sector	15	12.3
	Not working	98	80.3
Residence	Rural	16	13.1
	Urban	106	86.9
Smoking	Yes	1	0.8
	No	121	99.2

The mean gestational age was 29.39 weeks (SD = 9.55). Most of the participants (81.1%) had antenatal follow-up more than four times. Half of them (50%) had been pregnant three or more times, (33.66%) were nullipara and (44.3%) had experienced a miscarriage. Approximately (9%) of the participants experienced preeclampsia and (5.7%) had a family history of preeclampsia. More than half of them (59.8%) had no medical diseases, while (24.6%) had gestational diabetes (Table 2).

**Table 2:** Obstetrics and Medical Characteristics of the Participants.

Variables	*Mean*	*SD*	
Gestational age (in weeks)	29.39	9.554	
**Variables**		***N***	***%***
Antenatal visits	One	5	4.1
	Two	8	6.6
	Three	10	8.2
	Four or more	99	81.1
Number of pregnancies	One	34	27.9
	Two	27	22.1
	Three or more	61	50
Number of children	One	33	27
	Two	22	18
	Three or more	26	21.3
	None	41	33.6
Miscarriage	Yes	54	44.3
	No	68	55.7
Previous preeclampsia	Yes	11	9
	No	111	91
Family history of preeclampsia	Yes	7	5.7
	No	115	94.3
Medical disease	Hypertension	4	3.3
	Diabetes mellitus	8	6.6
	Gestational diabetes	30	24.6
	Hypothyroidism	3	2.5
	Asthma	1	0.8
	Multiple sclerosis	1	0.8
	Solitary kidney	1	0.8
	Hepatitis	1	0.8
	No	73	59.8

The total mean score for the knowledge scale was 25.43 (SD = 5.67). More than half of the participants (57.4%) reported having heard of preeclampsia before. The primary sources of information about preeclampsia were the internet/media (32.8%), doctors (31.4%), family/relatives (28.6%), books (4.3%), and nurses (2.9%). More than half of them (53.3%) identified high blood pressure as a sign of preeclampsia. However, they were less familiar with other signs and symptoms such as persistent headache (59.0%), edema (84.4%), blurred vision (61.5%), chest pain (82.0%), abdominal pain (60.7%), and back pain (68.0%). More than half of the participants were unaware of key preeclampsia risk factors, such as family history (62.3%), previous history of preeclampsia (50.8%), obesity (67.2%), diabetes mellitus (61.5%), unhealthy lifestyle (59.0%), multiple pregnancies (74.6%), and chronic hypertension (50.0%). More than half of them (55.7%) recognized fetal death as a complication of preeclampsia, while fewer were aware of other serious outcomes such as maternal death (28.7%), maternal heart disease (06.6%), and kidney dysfunction (22.1%) (Table 3).

**Table 3:** Knowledge of the Participants about Preeclampsia.

Item		*N*	*%*
Have you heard of preeclampsia before?	Yes	70	57.4
	No	52	42.6
What is your source of information about preeclampsia?	Doctors	22	31.4
	Nurses	2	2.9
	Family/relatives	20	28.6
	Internet/media	23	32.8
	Books	3	4.3
	**True**	**I don’t know**	**False**
**Signs/symptoms of preeclampsia**			
High blood pressure	65(53.3%)	55(45.1%)	2(01.6%)
Persistent headache	49(40.0%)	72(59.0%)	1(00.8%)
Edema	16(13.1%)	103(84.4%)	3(02.5%)
Blurred vision	46(37.7%)	75(61.5%)	1(00.8%)
Chest pain	15(12.3%)	100(82.0%)	7(05.7%)
Abdominal pain	44(36.1%)	74(60.7%)	4(03.3%)
Nausea and vomiting	58(47.5%)	63(51.6%)	1(00.8%)
Back pain	32(26.2%)	83(68.0%)	7(05.7%)
**Risk factors for preeclampsia**			
Family history of preeclampsia	36(29.5%)	76(62.3%)	10(08.2%)
Having prior preeclampsia	50(41.0%)	62(50.8%)	10(08.2%)
Obesity	29(23.8%)	82(67.2%)	11(09.0%)
Diabetes mellitus	40(32.8%)	75(61.5%)	7(05.7%)
Unhealthy lifestyle	46(37.7%)	72(59.0%)	4(03.3%)
Multiple pregnancies	21(17.2%)	91(74.6%)	10(08.2%)
Chronic hypertension	59(48.4%)	61(50.0%)	2(01.6%)
**Complications of preeclampsia**			
Maternal death	35(28.7%)	75(61.5%)	12(09.8%)
Fetal death	68(55.7%)	50(41%)	4(03.3%)
Maternal heart disease	8(06.6%)	107(87.7%)	7(05.7%)
Kidney dysfunction	27(22.1%)	89(73.0%)	6(04.9%)

Most of the participants outlined that they would seek medical care for signs such as diminution of fetal movement (96.7%), vaginal bleeding (97.5%), and blurred vision (92.6%). However, in cases of headache (39.3%), dizziness (18.0%), nausea (31.1%), and epigastric pain (23.0%) they would medicate themselves (Table 4).

**Table 4:** Attitude toward Preeclampsia Danger Signs.

Attitude	Seek medical care N (%)	Do nothing N (%)	Self-medication N (%)
Headache	62(50.8%)	12(09.8%)	48(39.3%)
Dizziness	88(72.1%)	12(09.8%)	22(18.0%)
Nausea	69(56.6%)	15(12.3%)	38(31.1%)
Epigastric pain	80(65.6%)	14(11.5%)	28(23.0%)
Diminution of fetal movement	118(96.7%)	4(03.3%)	0(00.0%)
Vaginal bleeding	119(97.5%)	3(02.5%)	0(00.0%)
Blurring of vision	113(92.6%)	5(04.1%)	4(03.3%)

The total mean score for the attitude scale was 23.39 (SD = 3.11). Most participants (85.2% and 83.6%) respectively agreed that early healthcare-seeking and regular antenatal care follow-up can reduce the complications related to preeclampsia. Furthermore, (48.4% and 80.3% respectively) agreed that avoiding stress and alcohol reduces the risk of preeclampsia. Additionally, (65.6% and 54.1% respectively) believed that increasing fruit and vegetable intake and reducing coffee intake can decrease the risk of preeclampsia. Significant numbers of the participants were unsure whether reduced urine output (58.2%) and convulsions (47.5%) were consequences of preeclampsia (Table 5).

**Table 5:** Attitude toward Preeclampsia.

Item	Agree N (%)	Neutral N (%)	Disagree N (%)
Avoiding stress reduces the risk of preeclampsia	59(48.4%)	47(38.5%)	16(13.1%)
Regular antenatal care can reduce the risk of preeclampsia	102(83.6%)	14(11.5%)	6(04.9%)
Fruit and vegetable intake can reduce the risk of preeclampsia	80(65.6%)	35(28.7%)	7(05.7%)
Reducing coffee consumption decreases the risk of preeclampsia	66(54.1%)	47(38.5%)	9(07.4%)
Avoiding alcohol consumption reduces the risk of preeclampsia	98(80.3%)	20(16.4%)	4(03.3%)
Preeclampsia is dangerous for both mother and fetus	106(86.9%)	13(10.7%)	3(02.5%)
Early health-seeking related to preeclampsia reduces its complication	104(85.2%)	17(13.9%)	1(00.8%)
Convulsion is a consequence of preeclampsia	57(46.7%)	58(47.5%)	7(5.7%)
Reduced urine output is a consequence of preeclampsia	45(36.9%)	71(58.2%)	6(04.9%)

A Pearson correlation coefficient was calculated to assess the association between participants’ mean total knowledge score and their total attitude score towards preeclampsia. A weak positive correlation was observed (r =.187, P =.040), indicating a statistically significant but weak association between knowledge and attitude about preeclampsia (Table 6).

**Table 6:** Correlation between Knowledge and Attitude of the Participants about Preeclampsia.

Correlations			
		Knowledge mean	Attitude mean
Knowledge mean	Pearson Correlation	1	.187*
	Sig. (2-tailed)		.040
	N	122	122
Attitude mean	Pearson Correlation	.187*	1
	Sig. (2-tailed)	.040	
	N	122	122

*Correlation is significant at the 0.05 level (2-tailed).

Further analysis revealed significant associations between participants’ knowledge of preeclampsia and educational attainment (p < 0.001) and employment status (p = 0.007). Participants with higher education levels demonstrated greater knowledge, with those holding postgraduate degrees achieving the highest mean score 32.29 (SD = 5.94), compared to college graduates 25.89 (SD = 5.33) and those with a high school education or less 23.67 (SD = 5.30). Similarly, employed participants had higher knowledge scores of 28.21 (SD = 5.63) than unemployed participants 24.76 (SD = 5.50) (Table 7).

**Table 7:** The Association between Knowledge of Participants about Preeclampsia and Their Sociodemographic Background.

	Knowledge Level		
**Variable**	**Pearson Correlation (r)**	**N**	**P-value**
**Age**	0.092	122	0.315
	**Mean**	**SD**	**P-value**
**BMI**			0.474
Underweight	22.33	2.51	
Proper weight	25.10	5.89	
Overweight	26.31	6.13	
Obese	24.72	5.39	
**Educational level**			<0.0001*
High school or less	23.67	5.30	
College graduate	25.89	5.33	
Postgraduate degree	32.29	5.94	
**Employment**			0.007*
Yes	28.21	5.63	
No	24.76	5.50	
**Employment sector**			0.417
Health sector	29.44	6.69	
Non-health sector	27.46	4.99	
**Residence**			0.709
Rural	24.93	5.29	
Urban	25.50	5.74	

*Significant association

Regarding attitudes towards preeclampsia, no statistically significant associations were observed for age (r = 0.081; p = 0.322), BMI (p = 0.485), educational level (p = 0.947), employment status (p = 0.446), and residence (p = 0.377) (Table 8).

**Table 8:** The Association between Attitude of Participants about Preeclampsia and Their Sociodemographic Background.

	Pearson Correlation (r)		
**Variable**	**Pearson Correlation (r)**	**N**	**P-value**
**Age**	0.081	122	0.322
	**Mean**	***SD***	**P-value**
**BMI**			0.485
Underweight	23.75	2.95	
Proper weight	25.00	2.65	
Overweight	22.91	2.89	
Obese	23.62	3.55	
**Educational level**			0.947
High school or less	23.51	3.75	
College graduate	23.31	2.69	
Postgraduate degree	23.42	2.76	
**Employment**			0.446
Yes	22.95	3.23	
No	23.50	3.08	
**Employment sector**			0.496
Health sector	23.55	3.00	
Non-health sector	22.60	3.41	
**Residence**			0.377
Rural	22.75	4.38	
Urban	23.49	2.88	

## Discussion

This study utilized a quantitative cross-sectional design conducted at the Obstetrics and Gynecology Outpatient Department of King Abdulaziz Medical City in Saudi Arabia. The study surveyed 122 pregnant women to assess their knowledge and attitudes toward preeclampsia. The mean knowledge score in this study was 25.43 (SD = 5.67), approximately 67% of the maximum score (38) suggesting that many participants demonstrated a moderate understanding of preeclampsia. This contrasts with Radwan et al.‘s [21] findings, where 49.4% of Saudi women of reproductive age were classified as having inadequate knowledge about preeclampsia. The discrepancy may reflect differences in study populations, as women in this study may have greater exposure to information due to higher education levels, younger age, and primarily urban residency, whereas Radwan et al.’s study included a broader demographic. Additional factors, such as sample size, data collection methods, and inclusion criteria, may also contribute to the observed differences.

Additionally, over half of our study’s participants recognized high blood pressure as a sign of preeclampsia but showed limited awareness of other symptoms like persistent headache, edema, blurred vision, chest and abdominal pain, nausea, vomiting, and back pain. This finding aligns with the studies by Gari et al. [14] and Fondjo et al. [20], which also showed high blood pressure as a commonly identified symptom of preeclampsia among pregnant women in Kumasi-Ghana and Saudi Arabia, respectively.

Furthermore, our results indicated that chronic hypertension and previous history of preeclampsia were the most acknowledged risk factors among participants, consistent with Gari et al. [14]. However, about 70% of the participants in this study were either overweight or obese which indicates a critical gap in addressing weight management as a modifiable risk factor. This underscores the need for educational interventions to focus more on obesity prevention and management during pregnancy. Moreover, comprehensive maternal health care education should cover all symptoms of preeclampsia to enable effective monitoring and early intervention.

In terms of complications, more than half of the participants were aware of fetal death because of preeclampsia, but fewer recognized maternal death, heart disease, and kidney dysfunction. This mirrors findings from Radwan et al. [21], where a significant number of the participants identified fetal death as a major complication, indicating a focus on neonatal outcomes over maternal health.

The primary sources of information about preeclampsia in our study were the internet/media, doctors, and family, with nurses being the least consulted. This contrasts with Jama [11] and Romuald et al. [19], where participants frequently cited midwives, obstetricians, and nurses as sources of preeclampsia education. This difference likely reflects cultural variation including limited access to midwife-led care in the region.

In our study, participants demonstrated a mean attitude score of 23.39 (SD = 3.11), placing the average participant at approximately 87% of the maximum score (27). This suggests that participants generally held favorable attitudes toward the risk factors, prevention, and complications of preeclampsia. This differs significantly from Mekie et al. [18], who reported that only 29.3% of the participants in South Gondar, Northwest Ethiopia, had a positive attitude towards preeclampsia, with 64.4% neutral and 6.3% negative. The variation in attitudes between the two studies may be partially attributed to differences in data collection methods, where face-to-face interviews may have influenced responses, leading to lower reported positive attitudes in Mekie et al.‘s study.

Most of our participants indicated they would seek medical care for symptoms such as reduced fetal movement, vaginal bleeding, and blurred vision. However, many would self-medicate for headaches, dizziness, nausea, and epigastric pain, possibly perceiving these symptoms as normal physiological changes during pregnancy. In contrast, a study by Romuald et al. [19] found that while most participants would seek medical care for various preeclampsia danger signs, a small percentage would either self-medicate for headaches or do nothing for edema. Additionally, Jama [11] reported that 40% of the participants preferred resting at home for symptoms like leg edema and constant headache.

A significant number of participants in our study recognized the importance of early health-seeking and regular antenatal care follow-up in reducing preeclampsia complications. However, about one-half were unsure if reduced urine output and convulsions were consequences of preeclampsia. Mekie et al. [18] reported similar findings regarding the importance of early health-seeking and regular antenatal care but noted less certainty about the significance of reduced urine output and convulsions among their participants.

Our study found a weak but significant positive correlation between participants’ total knowledge and attitude scores, suggesting that higher knowledge levels are associated with more positive attitudes toward preeclampsia. However, this relationship was not strong. This contrasts with the findings of Sangeetha and Baby [22], who reported a stronger correlation, indicating that increased knowledge about preeclampsia significantly improves both attitude and practice.

In this study, educational level and employment status significantly influenced participants’ knowledge of preeclampsia, with higher education levels associated with greater awareness. In comparison, Osman et al. [23] in the Al Baha region, Saudi Arabia, identified a history of preeclampsia as a key factor contributing to higher knowledge levels among women.

Interestingly, no statistically significant associations were found between participants’ attitudes toward preeclampsia and their sociodemographic characteristics. While participants with proper weight showed slightly higher attitude scores compared to others, this difference did not reach statistical significance. Similar findings were reported in previous studies [11,18,19], where no significant associations between attitudes and demographic characteristics were observed.

## Limitations

Limitations of this study include the small sample size which may restrict the generalizability of the findings. Additionally, the use of non-probability sampling methods and the exclusion of non-literate participants could introduce selection bias, potentially affecting the representativeness of the results

## Recommendations

This study recommends diverse sampling methods in future research to ensure a broader, more socio-demographically diverse sample. Conducting research in midwife clinics is also suggested to explore their influence on women’s knowledge of preeclampsia. Emphasis should be placed on educating women through integrated sessions during routine antenatal visits, led by midwives who can also utilize social media and community events. Key strategies include distributing informational leaflets, organizing educational classes in community health centers, and employing social media to enhance awareness. To further improve outcomes, programs should incorporate practical strategies for achieving healthy weight management, with regular discussions between healthcare providers and patients about the importance of weight control in reducing preeclampsia risk. Integrating these measures into antenatal care could significantly reduce the incidence of preeclampsia and associated maternal and neonatal complications.

## Conclusion

The study reveals a moderate understanding of preeclampsia and a generally favorable attitude among pregnant women in Saudi Arabia. However, significant gaps in recognizing critical symptoms and complications highlight the need for enhanced prenatal education to improve awareness and promote early detection, thereby reducing maternal and neonatal risks. Targeted public health initiatives and prioritizing education, particularly for high-risk groups, are essential for effective management of preeclampsia. This study contributes to the existing body of literature by providing specific insights into the knowledge and attitudes toward preeclampsia among pregnant women in a region where such data was previously scarce. The strength of this study lies in its focused demographic and geographical context, adding valuable data that can inform targeted educational interventions and improve maternal health outcomes in similar settings.

## Supporting information

S1 Table Table of the Included and Excluded Studies. (DOCX)
