# Supplementary material for: Knowledge and attitude of pregnant women about preeclampsia in King Abdulaziz Medical City, Western Region: A cross-sectional study
Source: PLoS One. 2025 May 7;20(5):e0312304. doi: 10.1371/journal.pone.0312304 (PMC12058169; doi:10.1371/journal.pone.0312304)
Supplement: S1 Table — (DOCX) [file pone.0312304.s001.docx]

**Table of the Included and Excluded Studies**

**Included studies:**

| **No.** | **Study tittle** | **Year** | **Authors** | **Study aim** |
| --- | --- | --- | --- | --- |
| 1 | Knowledge and attitude of pregnant women towards preeclampsia and its associated factors in South Gondar Zone,  Northwest Ethiopia: a multi‐center facility‐based cross‐sectional study | 2021 | Maru Mekie, Dagne Addisu  , Minale Bezie  , Abenezer Melkie  , Dejen Getaneh  , Wubet Alebachew Bayih and  Wubet Taklual | To assess the knowledge and attitude towards preeclampsia and its associated  factors in South Gondar, Northwest Ethiopia. |
| 2 | Knowledge of preeclampsia and its  associated factors among pregnant  women: a possible link to reduce related  adverse outcomes | 2019 | Linda A. Fondjo , Vivian E. Boamah  , Adelaide Fierti  , Dorcas Gyesi and Eddie-Williams Owiredu | To assess the level of knowledge of preeclampsia and evaluated the factors associated with knowledge adequacy among  pregnant women attending antenatal care at a University Hospital in Kumasi-Ghana. |
| 3 | Level of knowledge on  preeclampsia symptoms,  complications, and risk factors  among women in Saudi Arabia:  A cross sectional study | 2022 | Abdulrahim Gari,  , Wafa Alshanqiti  , Fatimah  Alshanqiti  , Rehab Alquzi  , Rawan Alsamli  , Reem Alqahtani | To determine the state of  knowledge of preeclampsia among women who live in Makkah, Saudi Arabia. |
| 4 | The knowledge and attitude towards  preeclampsia among pregnant women  attending banadir and medina hospitals in  mogadishu-somalia | 2019 | Abdinasir Abdullahi Jama | To explore the knowledge and attitude towards preeclampsia  among pregnant women attending Banadir and Medina hospitals in Mogadishu-Somali. |
| 5 | The women knowledge, attitude, and perceptions of pre-eclampsia and eclampsia in Madagascar | 2019 | Randriamahavonjy Romuald, Tanjona A. Ratsiatosika, Rakotonirina A. Martial, Rainibarijaona A. Lantonirina, Rakotonirina Ando-Miora, Andrianampanalinarivo H. Rakotovao | To assess patients' knowledge, attitudes and beliefs about pre-eclampsia in Madagascar. |
| 6 | Preeclampsia knowledge among postpartum women treated for preeclampsia and eclampsia at Korle Bu Teaching Hospital in Accra, Ghana. | 2020 | Avina Joshi, Titus K. Beyuo, Samuel A. Oppong, Cheryl A. Moyer, Emma R. Lawrence | To explore preeclampsia knowledge among postpartum women treated for preeclampsia and eclampsia at Korle Bu Teaching Hospital in Accra, Ghana. |
| 7 | Women´s experiences of preeclampsia  as a condition of uncertainty: a qualitative study | 2022 | Therése Hansson, Maria E. Andersson, Gerd Ahlström and Stefan R. Hansson | To describe women´s experiences of preeclampsia to improve the support and care  given during and after pregnancy. |
| 8 | Epidemiological trends of maternal  hypertensive disorders of pregnancy at the global, regional, and national levels: a population‐based study | 2021 | Wei Wang, Xin Xie  , Ting Yuan, Yanyan Wang  , Fei Zhao, Zhangjian Zhou and Hao Zhang | To determine the  epidemiological characteristics of hypertensive disorders of pregnancy in a populationbased cohort. |
| 9 | Prevalence, risk factors, and fetal and maternal outcomes of hypertensive disorders of pregnancy: a retrospective  study in western saudi arabia | 2018 | Ahmed Hussein Subki, Mohammed Ridha Algethami, Wejdan  Mohammad Baabdullah, Majed Nasser Alnefaie, Mashael Abdullah  Alzanbagi, Rawan Marzooq Alsolami and Hassan S. Abduljabbar | To estimate the prevalence of hypertensive disorders of pregnancy  (HDP) in Saudi Arabia as well as the risk factors of HDP, and maternal and fetal  outcomes. |
| 10 | Vegetable dietary pattern associated with low risk of preeclampsia possibly through reducing proteinuria | 2019 | Baibing Mi , Xiaozhong Wen , Shanshan Li , Danmeng Liu , Fangliang Lei , Ruru Liu , Yuan Shen , Yue Chen , Lingxia Zeng , Xin Liu , Shaonong Dang , Hong Yan | To examine the associations between dietary patterns during pregnancy and the risk of preeclampsia. |
| 11 | Global and regional estimates of preeclampsia and eclampsia: a systematic review. | 2013 | Edgardo Abalos, Cristina Cuestaa, Ana L. Grosso, Doris Chou, Lale Say | To evaluate hypertensive disorders of pregnancy magnitude globally and in different regions and settings. |
| 12 | The effects of an educational programme about preeclampsia on women’s awareness: a randomised control trial. | 2020 | K. Alnuaimi MSc, BSc, PhD, J. Abuidhail RN, PhD, H. Ismail RN, MSN | To examine the effects of an interventional programme about preeclampsia on high-risk preeclampsia Jordanian women’s awareness and pregnancy outcomes. |
| 13 | Preeclampsia: clinical features and diagnosis. | 2022 | Phyllis August, MD, MPHBaha M Sibai, MD | To discuss the clinical features, diagnosis, and differential diagnosis of preeclampsia. |
| 14 | Perspectives, preferences and needs regarding early prediction of preeclampsia in Dutch pregnant women: A qualitative study. | 2017 | Neeltje M. T. H. Crombag, Marije Lamain-de Ruiter, Anneke Kwee, Peter C. J. I. Schielen, Jozien M. Bensing, Gerard H. A. Visser, Arie Franx & Maria P. H. Koster | To explore pregnant women’s perceptions, preferences and needs regarding prediction models for first trimester screening for common pregnancy complications, such as preeclampsia, to support future implementation. |
| 15 | Prevention of Preeclampsia | 2012 | Sammya Bezerra Maia e Holanda Moura,Laudelino Marques Lopes,  Padma Murthi, and Fabricio da Silva Costa | To review this  recent evidence on the primary and secondary prevention of  preeclampsia. |
| 16 | Preeclampsia and Eclampsia | 2017 | National institute of child health and human development. | N/A |
| 17 | Awareness of preeclampsia and its associated factors among women in Al Baha region, Saudi Arabia. | 2023 | Tajelsir Osman, Eman A. Keshk, Abdullah Ali S. Alghamdi, Mohammed Ahmed A. Alghamdi, Mohammed Abdullah A. Alghamdi, Ahmed A. Alzahrani, Khalid N. Alghamdi, Yasser A. Alzahrani, Abdulrahman A. Alghamdi, Rahaf A. Alghamdi | To evaluate the awareness of preeclampsia and its associated factors among women in the Al Baha region, Saudi Arabia. |
| 18 | Awareness and knowledge of pre-eclampsia among Saudi women of reproductive age. | 2023 | Ashraf Radwan, Manar Al Naji, Nourah Alyoubi, Iram Alsallat, Zakeiah Alsulaimani, Shaima Ali Albeladi, Hussein Sabban, Abdulmageed Abdou, Ali Alsamry | To assess pre-eclampsia understanding among Saudi women of reproductive age. |
| 19 | Statistical yearbook of Saudi ministry of health. | 2022 | Saudi Ministry of Health | N/A |
| 20 | Assessment of knowledge, practice and attitudes regarding preeclampsia among women with pregnancy induced hypertension at selected hospital, Bangalore | 2018 | Sangeetha C, Baby Prasnna | 1. To assesses the Knowledge, attitude and practice of preeclampsia among PIH women. 2. To correlate between knowledge, practice and attitude on women with pregnancy induced hypertension. 3. To associate knowledge, attitude and practice score with selected demographic variables. |
| 21 | Netter’s Obstetrics and Gynecology E-Book. Elsevier | 2023 | Smith, R. P. | N/A |
| 22 | Maternal mortality. | 2019 | World Health Organization. | N/A |
| 23 | Association between mental stress and gestational hypertension/preeclampsia. | 2013 | S Zhang, Z Ding, H Liu, Z Chen, J Wu, Y Zhang, Y Yu | To evaluate the relationships between mental stress and gestational hypertension/preeclampsia in pregnant women. |

**Excluded studies:**

| **No.** | **Study tittle** | **Study year** | **Authors** | **Study aim** | **Reason for exclusion** |
| --- | --- | --- | --- | --- | --- |
| 1 | Content Analysis of Patient-Facing Information Related to  Preeclampsia | 2022 | Kimberley H. Geissler, PhD, Valerie Evans, MSc, Michael I. Cooper, BBA,  Susan J. Shaw, PhD, Christina Yarrington, MD, FACOG, Laura B. Attanasio, PhD | To examine commonly used preeclampsia  information sources to evaluate whether pregnant people are receiving up-to-date, guideline-based information. | Irrelevant to the main variables under study. |
| 2 | Knowledge about Preeclampsia among Women of Reproductive  Age Attending Al-BeydaMedical Center | 2022 | Faiza Taher, Marfoua Ali, Ghada Otman | To assess the knowledge of preeclampsia and it is dangerous signs and maternal  and fetal complications, among women of reproductive age attending Albeyda Medical Center. | This study has repetitive information that already included from other literature review. |
| 3 | Resident physicians’ and midwives’ knowledge of preeclampsia and eclampsia  reflected in their practice at a clinical hospital in southern romania | 2019 | Carmen Liliana Soggiu-Duta, Nicolae Suciu | To assess the current state of resident physicians’ and midwives’ knowledge of preeclampsia and eclampsia reflected in their practice at  a clinical hospital in southern Romania. | Different type of population. |
